# How is patient-centred care conceptualized in women’s health: a scoping review

**DOI:** 10.1186/s12905-019-0852-9

**Published:** 2019-12-10

**Authors:** Anna R. Gagliardi, Bryanna B. Nyhof, Sheila Dunn, Sherry L. Grace, Courtney Green, Donna E. Stewart, Frances C. Wright

**Affiliations:** 1 Toronto General Hospital Research Institute, University Health Network, 200 Elizabeth Street, 13 EN-228, Toronto, ON M5G2C4 Canada; 2 Toronto General Hospital Research Institute, University Health Network, Toronto, Canada; 3 Women’s College Hospital, Toronto, Canada; 4 York University and University Health Network, Toronto, Canada; 5 Society of Obstetricians and Gynaecologists of Canada, Ottawa, Canada; 6 Sunnybrook Health Sciences Centre, Toronto, Ontario, Canada

**Keywords:** Women’s health, Patient-centred care, Equity, Quality, Frameworks, Models, Scoping review

## Abstract

**Background:**

Gendered disparities in health care delivery and outcomes are an international problem. Patient-centred care (PCC) improves patient and health system outcomes, and is widely advocated to reduce inequities. The purpose of this study was to review published research for frameworks of patient-centred care for women (PCCW) that could serve as the basis for quality improvement.

**Methods:**

A scoping review was conducted by searching MEDLINE, EMBASE, CINAHL, SCOPUS, Cochrane Library, and Joanna Briggs index for English-language quantitative or qualitative studies published from 2008 to 2018 that included at least 50% women aged 18 years or greater and employed or generated a PCCW framework. Findings were analyzed using a 6-domain PCC framework, and reported using summary statistics and narrative descriptions.

**Results:**

A total of 9267 studies were identified, 6670 were unique, 6610 titles were excluded upon title/abstract screening, and 11 were deemed eligible from among 60 full-text articles reviewed. None were based on or generated a PCCW framework, included solely women, or analyzed or reported findings by gender. All studies explored or described PCC components through qualitative research or surveys. None of the studies addressed all 6 domains of an established PCC framework; however, additional PCC elements emerged in 9 of 11 studies including timely responses, flexible scheduling, and humanized management, meaning tailoring communication and treatment to individual needs and preferences. There were no differences in PCC domains between studies comprised primarily of women and other studies.

**Conclusions:**

Given the paucity of research on PCCW, primary research is needed to generate knowledge about PCCW processes, facilitators, challenges, interventions and impacts, which may give rise to a PCCW framework that could be used to plan, deliver, evaluate and improve PCCW.

## Background

Inequities in access to and quality of health care are pervasive, leading to disparities in health outcomes. While there are multiple causal factors, one of the key issues is gender bias [1].

For example, research on access to care for cardiovascular disease revealed that women were far less likely to be referred for diagnostic tests and to cardiac rehabilitation compared with men [2], and even when referred, they were less likely to receive recommended treatment compared with men [3]. Similarly, another study of patients with acute myocardial infarction revealed that women received guideline-recommended interventions such as timely reperfusion, antiplatelet therapy, statins, and cardiac rehabilitation less often than men [4]. Such disparities in the quality of care for women may be heightened by race or ethnicity in both high- and low-resource countries, and by a lack of primary research including women participants [5, 6].

In 1995, the Fourth World Conference on Women of the United Nations highlighted the need to deliver health care services that are sensitive to the needs and preferences of women [2], and in 2009 the World Health Organization report, Women and Health, emphasized the need to improve the quality of women’s health care services [7]. Over time, the concept of women’s health has broadened from a focus on sexual and reproductive health to a life-course approach that considers other health challenges that affect women during and beyond their reproductive years, and the impact of social determinants on women’s health, morbidity and mortality [8]. As a result of ongoing gender bias that influences women’s health care and outcomes, one of the 17 goals in the United Nations report, Gender Equality in the 2030 Agenda for Sustainable Development, is to promote women’s health and well-being by ensuring that women have universal access to comprehensive health care that is responsive to gender and the life course [9].

Patient-centred care (PCC) is an approach that tailors care to individual needs, preferences and circumstances by informing, engaging, and empowering patients (including families or care partners) [10]. PCC is considered a key element of high quality health care because it has been associated with patient (knowledge, relationship with providers, service experience and satisfaction, treatment compliance, health outcomes) and health system (cost-effective service delivery) outcomes [11–13]. Accumulating research offers insight on the dimensions of PCC, but reveals discrepancies in what is thought to constitute PCC. A scoping review of 19 studies published from 1994 to 2011 identified 25 unique frameworks or models of PCC; common domains pertained to the patient-provider relationship (sharing information, empathy, empowerment), partnership (sensitivity to needs, relationship-building), and health promotion (collaboration, case management, resource use) [14]. A systematic review of 26 studies published from 1992 to 2008 identified 13 unique instruments to assess PCC, further underscoring variability in how PCC is conceptualized [15].

Unfortunately many patients do not receive PCC, particularly those with limited education, poor health, or whose culture differs from their health care provider. A national survey in the United States showed that, among 2718 responding adults aged 40 years or greater with 10 common medical conditions, there was considerable variation in whether patients were told they had a choice of treatment and whether they were asked for input in the decision [16]. In a study of 509 adult patients seen by family physicians or general internists, PCC was observed more in for healthier, more educated patients [17]. A survey of 80 providers and 27 Muslim women found that both groups identified similar barriers to PCC: providers lacked understanding of patients’ religious and cultural beliefs, and needs for modesty, and patients lacked understanding of disease processes and mistrusted the health care system [18].

Delivering patient-centred care for women (PCCW) may serve as an important means of reducing gendered disparities. However, it is unclear whether existing PCC frameworks or models are relevant to women’s health, or if the components, delivery and experience of PCCW varies among women with different health conditions or personal characteristics. Greater understanding is needed of what constitutes PCCW to support system-level planning of services for women, and to inform the development of interventions targeted to women and their care providers that support PCCW. While others have reviewed published research on frameworks or models of PCC [14], no one has synthesized knowledge about PCCW frameworks or models. The purpose of this study was to review published research, and identify and compare existing PCCW frameworks or models. If PCCW frameworks or models are available, they could serve as the basis for evaluating and improving care delivery and outcomes among women. If lacking, then primary research is needed to explore the elements of PCC valued by women to inform the development of a comprehensive PCCW framework.

## Methods

### Approach

For this study, a scoping review was chosen as the methodologic approach. A scoping review aims to rapidly map the key concepts in published research on a given topic [19]. Unlike a systematic review, which aims to provide answers to a well-defined research question, a scoping review addresses broader topics, includes research of various designs, describes the extent, range and nature of research, and identifies gaps in the existing literature [20]. A scoping review consists of five steps: scoping, searching, screening, data extraction, and data analysis. Reporting of the methods and findings was guided by the Preferred Reporting Items for Systematic Reviews and Meta-Analysis (PRISMA) criteria [21]. Data for this review were publicly available so institutional review board approval was not necessary. A protocol was not registered for this review.

### Scoping

To become familiar with this topic, a quick scan of relevant literature was undertaken by searching MEDLINE using the Medical Subject Headings: women’s health and patient-centered care. The titles and abstracts of the initial search results were screened by KB and ARG, and discussed by the research team to collectively establish eligibility criteria based on the Population, Issue, Comparisons, Outcomes (PICO) framework, which then informed the comprehensive search strategy. Given the preliminary nature of a scoping review, the research team decided to focus on general PCCW frameworks pertaining to women with any health care concern or condition, and to investigate disease-specific PCCW frameworks in a separate review (reported separately).

*Population* referred to women, family members or care partners aged 18 years or greater with any health care concern or condition, or clinicians (i.e. physicians, nurses) involved in the care of those women in any type of health care setting. *Issues* referred to the identification or development of PCCW frameworks in which PCC was explicitly labelled in the published manuscript as patient-, person-, woman-, women-, client- or family-centered/centred care. Although there is no standardized definition of PCC, PCC can be thought of as the individualized, timely, coordinated, respectful, and compassionate care of patients that engages women and takes into consideration their values, preferences, information and supportive care needs, such that they have the ability to make clinical decisions and manage their own health [10]. With respect to *comparisons*, qualitative (interviews, focus groups, qualitative case studies), quantitative (questionnaires, randomized controlled trials, time series, before/after studies, prospective or retrospective cohort studies, case control studies) or mixed methods studies were eligible if they explored and/or compared patient or clinician views about PCCW, experiences with PCCW including enablers or barriers, or suggestions for improving PCCW, or evaluated the impact of strategies implemented to support or improve PCCW. Such studies could evaluate PCCW in patients and/or clinicians with and without exposure to interventions, before and after exposure, or across different interventions. The primary *outcome* of interest was a PCCW framework (or model, theory, etc.), either employed by study, or compiled or developed by the study based on data collected in any of the aforementioned ways that may have included one or more of, but was not limited to PCCW constructs, processes, determinants (enablers, barriers), or outcomes.

Studies were considered ineligible if they primarily involved trainees (i.e. medical students or residents) or allied health care professionals (i.e. dentists, physiotherapists); concluded that PCC is needed without having studied PCC; labelled any form of clinical care or multi−/inter-disciplinary care for patients as PCC; investigated patient engagement in organizational- or system-level health service planning; examined the illness experience of patients rather than the care experience; focused on care delivered by peers or lay persons; or studied the patient-centered medical home, health-related quality of life, electronic applications for patients, or patient preferences for treatment or clinical outcomes. Publications in the form of protocols, editorials, commentaries, letters, or meeting abstracts or proceedings were excluded. Systematic reviews were also excluded although references were searched for eligible primary studies.

### Searching

A comprehensive literature search was conducted on February 26, 2018 in MEDLINE, EMBASE, SCOPUS and CINAHL by ARG based on a search strategy that was devised by a medical librarian and complied with the Peer Review of Electronic Search Strategies checklist (Additional File 1) [22]. The search was purposefully broad, including concepts for women’s health combined with explicit mention of PCC-related terms; by choosing to screen a large number of search results, we hoped to capture studies that generated frameworks that might otherwise be missed. The search was limited to research published in English language from 2008 to that date, a 10-year time span during which research on PCC became prevalent. We chose not to search the grey literature given the methodological challenges that have been identified by others such as sensitivity versus specificity, replicability, risk of bias, and intensity of time and effort [23, 24]. Search results were exported into Excel, in which screening and data extraction were performed.

### Screening

As a pilot test, KB, HL and ARG independently screened 50 records, and then compared and discussed findings to establish a shared understanding of how to interpret and apply eligibility criteria. Then titles and abstracts were screened independently by KB and HL. All articles considered potentially eligible by at least one reviewer were retrieved for full-text screening, which was undertaken concurrent with data extraction. During screening it became apparent that few studies focused on women only, so it was decided to include studies if at least half of the participants were women or outcomes were analyzed by gender.

### Data extraction

A data extraction table was developed to collect information on author, publication year, country, research design (including methods of data collection, number of participants, age range, proportion of female participants), study objective, term used to refer to PCC, definition or description of PCC employed or generated, and related findings. HL extracted data, which were independently checked by ARG.

### Data analysis

Summary statistics were used to report the number of studies published per year, country, research design, and term used for PCC. Definitions or descriptions of PCCW across all studies were reported textually. To further analyze and compare PCCW across studies, PCC elements employed or generated in each study were mapped to the McCormack et al. PCC framework, and the number of domains addressed in each study was summarized [25]. The McCormack et al. framework was chosen from among other PCC frameworks we identified in the literature because it was rigorously developed, extends beyond the clinical encounter, and is more comprehensive than other frameworks [14]. It was established by systematically reviewing literature and relevant theories, observing 38 medical encounters between cancer patients and oncologists, interviewing those 38 patients, and then reviewing the proposed domains with a 13-member expert panel to refine the framework. The resulting PCC framework consists of 31 sub-domains within six domains: fostering clinician-patient relationships, exchanging information, recognizing and responding to patient emotions, managing uncertainty, making decisions, and enabling patient self-management.

## Results

### Search results

**Figure 1:**
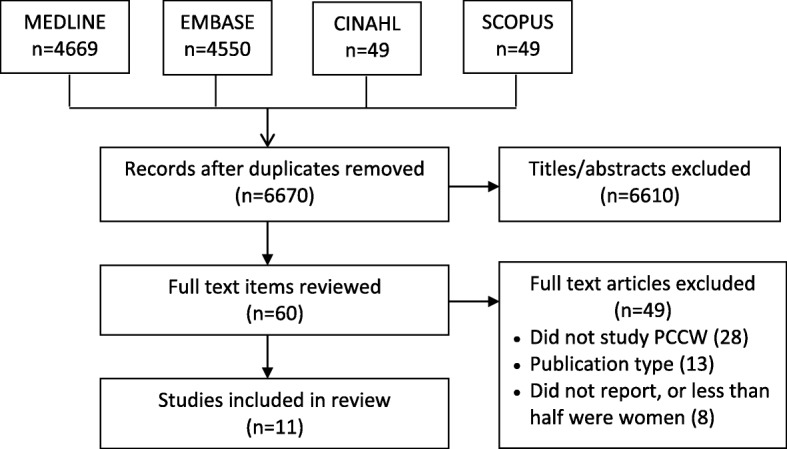
PRISMA diagram

A total of 9267 studies were identified, of which 6670 were unique, and 6610 titles were excluded, leaving 60 full-text articles to be screened. Of these, 48 articles were excluded: 28 did not study PCCW, 13 were not an eligible publication type, and 8 did not report the number of women participants or less than half were women. A total of 11 studies were eligible for review (Fig. 1). Data extracted from these studies are shown in Table 1.

**Table 2:** PCC domains measured or revealed in included studies

Study	Fostering the relationship	Exchanging information	Addressing emotions	Managing uncertainty	Making decisions	Enabling self-management	Domains per study (n)
	• Discuss roles and responsibilities • Honesty and openness • Trust in clinician competence • Express caring • Build rapport	• Explore needs and preferences • Share information • Provide information resources • Assess and facilitate understanding	• Explore and identify emotions • Assess anxiety or depression • Validate emotions • Express empathy or reassurance • Provide help to deal with emotions	• Define uncertainty • Assess uncertainty (cognitive) • Use emotion-focused management strategies (affective) • Use problem-focused management strategies (behavioural)	• Communicate about decision needs, support and process • Prepare for deliberation and decision • Make and implement a choice and action plan • Assess decision quality and reflect on choice	• Learn and assess • Share and advise • Prioritize and plan • Prepare, implement and assist • Arrange and follow-up	
Cheraghi 2017 Iran [26]	x	x	–	–	x	x	4
Cuevas 2017 United States [27]	–	x	–	–	x	–	2
Adamson 2017 Scotland [28]	x	x	–	–	x	–	3
Gill 2016 Canada [29]	x	–	–	–	x	–	2
Doubova 2016 Mexico [30]	–	x	–	–	–	–	1
Raja 2015 United States [31]	x	x	–	–	–	–	2
Leijen-Zeelenberg 2015 Netherlands [32]	–	x	x	–	–	–	2
Papp 2014 Hungary [33]	x	x	x	–	–	–	3
Marshall 2012 Australia [34]	x	–	–	–	x	–	2
Bann 2010 United States [35]	–	–	–	–	–	–	0
Davis 2008 Australia [36]	x	x	–	–	–	–	2
Studies including domains (n, % of 11)	7 (63.6)	9 (72.7)	2 (18.2)	0 (0.0)	5 (45.5)	1 (9.1)	

### Study characteristics

Studies were published from 2008 to 2017 inclusive in both higher- and lower-resource countries with differing health care systems including the United States (*n* = 3), Australia (*n* = 2) and one each in Canada, Hungary, Iran, Mexico, Netherlands and Scotland. PCC was studied for critical and intensive care, chronic conditions, older people, ear, nose and throat care, primary care, surgical inpatients, and complementary and alternative medicine. In terms of research design, the majority of studies employed qualitative interviews or focus groups (8, 72.7%), while 3 studies involved surveys (27.2%). The majority of studies used the term patient-centered or -centred or -centeredness (10, 90.9%). No studies included solely women, and no studies analyzed or reported findings by gender. At least 80.0% of participants were women in 4 of the 11 studies [31, 34–36].

### PCCW framework

None of the 11 studies was based upon, or generated a framework, model, theory or approach specific to PCCW. Instead they sought to identify and describe the components of PCC.

### PCC definition or measurement

Of the 11 included studies, 3 (27.3%) did not a priori define PCC. Among the 8 (72.7%) studies that defined PCC, 2 (25.0%) studies referred to it as accommodating user views in the design or evaluation of health services (system level), and 6 (75.0%) studies referred to it as valuing people as individuals, respecting their needs and values, and addressing those preferences in clinical decisions (patient level). One study asked patients what PCC meant to them and despite being unfamiliar with the word patient-centred, they were able to articulate that it meant they were involved in their care, attended to, and connected with their clinician [34]. All 11 studies described how PCC was measured; among the qualitative studies, participants were asked about their experiences of care and what they valued about their clinician, environment and information received. Quantitative studies asked participants about attributes of PCC including respect for patient values, engagement, quality of communication and relationship with care providers.

### PCC domains

Table 2 summarizes the mapping of PCC concepts in included studies to McCormack’s PCC framework [25]. None of the studies addressed all 6 domains. Although one study addressed 4 of 6 domains and two studies addressed 3 of 6 domains. The domains most frequently addressed by the 11 studies were exchanging information (*n* = 8), fostering a patient-clinician relationship (*n* = 7) and making decisions (*n* = 5). With respect to exchanging information, patients wanted clinicians to ask about their life circumstances and personal values, listen and acknowledge needs or concerns, accommodate questions, detail next steps or follow-up care, provide information about treatment options, and justify treatment prescribed when counter to patient preference [26–28, 30–33, 36]. Regarding fostering a patient-clinician relationship, patients wanted clinicians to be respectful, advocate for them, get to know them, share information about themselves, engage family members, express empathy, not rush them, and allow them to maintain dignity [26, 28, 29, 31, 33, 34]. Regarding decision-making, patients wanted to be sufficiently informed such that they and family members could be involved in decisions, which they said enhance satisfaction, respect, dignity [26–29, 34]. Domains least addressed among the 11 studies were responding to patient emotions (*n* = 1), enabling patient self-management (n = 1) and managing uncertainty (*n* = 0). Patients said that clinicians should offer more attention to emotional and psychological needs [32], and they appreciated autonomy when clinicians outlined self-care activities [26]. There were no differences in PCC domains between studies comprised primarily of women [31, 34–36] and other studies.

**Table 1:** Data extracted from included studies

Study	Research design	Objective	PCC label	PCC definition or measure	McCormack PCC elements described or recommended	Additional PCC elements described or recommended
Cheraghi 2017 Iran [26]	Qualitative interviews with patients (*n* = 10; mean age 52.6; 5 women, 5 men) 50.0% women	To explore and describe PCC in critical care units	patient-centered	Authors defined PCC as the provision of respectful care in response to patients’ preferences, needs and values During interviews, participants were asked to describe experiences and perceptions of PCC and how it can be achieved; themes established inductively	• Relationship: Maintaining human dignity, fulfilling patients’ needs • Information: Establishing therapeutic communication, analyzing the situation, individualizing care; patients receiving adequate information about treatment reduced anxiety • Decision-making: Involving patients in decision-making increased satisfaction with care • Self-management: Clinicians addressed and alleviated concerns, and outlined self-care activities, which promoted patient autonomy	• Ease of contact: Timely response to requests gained patient trust and sense of security • Humanization: identify, prioritize, and fulfill patients’ biological, psychosocial, and spiritual needs), individualization of care
Cuevas 2017 USA [27]	Qualitative focus groups with patients (*n* = 142; age not reported; 14 groups with women, 13 with men; n in each group not reported) 51.9% women groups	To explore views about PCC across three groups with chronic conditions (i.e. diabetes, hypertension) in primary care: African Americans, Europeans and Latinos	patient-centered	PCC not defined During interviews, participants were asked about what makes a good and bad experience; themes established inductively	• Information: Patients want clinician attentive to patients’ needs and listen to their comments/ concerns • Decision-making: Patients wanted to be more participatory in their interactions with providers and be more involved in their own care	• Consider race/ethnicity: African Americans felt that it was important for clinician to consider patient’s race in treatment plans • Speak native language: Many preferred a physician that knew their language in order to communicate effectively with patients, enable patients to understand their recommendations • *differences in findings between men and women not reported
Adamson 2016 Scotland [28]	Qualitative interviews with patients (*n* = 15, aged 69 to 95; 8 women, 7 men) 53.3% women	To understand the meaning of PCC for older people attending day hospitals for a variety of health care issues	person-centred	PCC not defined During interviews, participants were asked about talking with nurses, relationships, involvement in decisions, feeling valued, and getting information; themes emerged inductively	• Relationships: Developed trusting relationship with nursing staff, depended on nurses and had confidence that they would advocate for them, were informed about their progress with treatment or care, built rapport with staff • Information: patients appreciated when they were informed about how they were progressing with treatment. Following treatment, patients valued knowing what was coming next in care. • Relationships: when staff shared aspects of their own life with the patient and participated in casual ‘banter’ this strengthened the relationship with clinical staff • Decision-making: Patients felt involved in decision-making, which enhanced their dignity and respect	• Coordination of care: Patients reported that they were seen by clinicians in their home following treatment to see if they had everything they needed. This continuation of care was appreciated
Gill 2016 Canada [29]	Qualitative interviews with patients and family members (*n* = 32; 15/46.9% aged 50 to 64, 8 were < 50, 9 ≥ 65; 11 patients, 21 family; 17 women, 15 men) 53.1% women	To understand views about PCC among intensive care unit patients and their families	patient-centered	PCC not defined During interviews participants were asked to describe experiences they wished had been different; themes emerged inductively	• Decision-making: Family felt stressed about being patient’s spokesperson. Families’ ability to make decisions about patient care and have confidence in their decisions was impacted by the information and support they received. • Relationship: Providers were perceived as impatient and family members sometimes felt dismissed; they desired greater empathy	–
Doubova 2016 Mexico [30]	Telephone survey of patients (*n* = 6005; 82.2% were aged 20 to 59; 3126 women, 2869 men) 52.1% women	To explore public views about the PCC elements that contribute to high quality primary care	patient-centered	PCC takes into account the view of users in the design, provision and evaluation of health care services Survey included 10 PCC attributes based on Commonwealth Fund survey used in other countries: primary care provider (PCP) knows relevant information about the patient’s medical history; PCP gives an opportunity to ask questions about recommended treatment; PCP spends enough time with the patient; PCP explains things in a way that is easy to understand; PCP helps the patient to coordinate or arrange his/her healthcare from other doctors and places; patient perceives difficulties in communication with the primary care clinic during regular practice hours about a health problem; a nurse or another clinical staff (other than a doctor) is involved in primary healthcare; PCP who during a routine medical checkup in the past 2 years talked about an exercise or physical activity; PCP who during a routine medical checkup in the past 2 years spoke of a healthy diet and healthy eating; PCP who during a routine medical checkup in the past 2 years talked about things that worry the patient or cause stress	• Information: PCP provides information and explanations, and opportunities to ask questions	• Ease of contact: Easy to reach the primary care clinic • Problems are solved: PCP solves most health problems • Familiarity with patient: PCP knows relevant info about patient’s medical history • Coordination of care: PCP coordinates healthcare
Raja 2015 USA [31]	Qualitative interviews with patients (*n* = 20; aged 21 to 74; 18 women, 2 men) 90.0% women	To explore views about PCC among primary care patients with little or no health insurance	patient-centered	Authors cited Institute of Medicine PCC definition: Providing care that is respectful and responsive to individual patient preferences, needs, and values, and ensuring that patient values guide all clinical decisions During interviews, participants were asked about what made visits positive or negative; themes emerged inductively	• Relationship: Chatting with patients, asking questions, and telling patients about themselves helps build rapport • Information: Give overview of the procedure and clear expectations, results, appointment flow, realistic expectation of pain; patients desired more time with provider	• Physical setting: Pleasant environment makes participants feel respected and welcomed • Humanization: Feeling listened to, cared for, or seen as an entire human being with needs and emotions; providers consider the totality of a patient’s physical health, their ways of coping, and their environment • Avoiding jargon: Express technical terms in an understandable manner • Ease of contact: Inability to schedule appointments led to feeling devalued
Leijen-Zeelenberg 2015 Netherlands [32]	Qualitative interviews with patients (*n* = 22; mean age 52.8 years; 13 women, 9 men) 59.1% women	To explore PCC views and preferences among those visiting an ear, nose & throat outpatient unit	patient-centred	Authors defined PCC using Institute of Medicine (IOM) 6 domains: Respect for patients’ values, preferences and expressed needs; Information, communication and education; Coordination and integration of care; Emotional support - relieving fear and anxiety; Physical comfort; Involvement of family and friends During interviews, participants were asked to share experiences related to each of the 6 IOM domains; views about themes emerged inductively	• Information: Some respondents felt that they had to be assertive at the clinic in order to get respect for their preferences and needs. Being able to ask questions during consultations and receive clear responses was very important. It is also important to get an explanation when an expressed preference is not being complied with. • Emotional support: More attention needed on emotional and psychological support.	• Coordination of care: some find it difficult to plan more than 3 months in advance and disliked the inflexibility in the planning system. Negative experiences due to alternating doctors (seeing different doctors at subsequent appointments). • Physical setting: Nice atmosphere at outpatient clinic helps provide physical comfort. • Involvement of family and friends
Papp 2014 Hungary [33]	Qualitative focus groups (*n* = 61; 14 groups with 8 to 10 per group; 69.8% aged 41 or greater; 34 women, 27 men) 55.7% women	To explore views about the elements of high quality primary care including patient-centeredness	patient-centered	Authors defined PCC as the degree to which a system actually functions by placing the patient at the center of its delivery of health-care, assessed in terms of patient’s experience During interviews, participants were asked about general aspects of quality and elements of patient-centeredness; themes emerged inductively	• Information: Nurses should have an important role in providing information to patients; physicians should spend time to explain the situation to patients • Relationship: Patients expect doctor to be empathetic, friendly, attentive, good listeners, sympathetic, and willing to help	• Avoiding jargon: Information for patients should be understandable and clear • Humanization: Patients expect to be treated as a human being, not only as a disease
Marshall 2012 Australia [34]	Qualitative interviews with patients (n = 10; aged 30 to late 60’s; 8 women, 2 men) 80.0% women	To explore views about PCC among surgical inpatients	patient-centred	Authors note inconsistency in PCC definitions (treating people as individuals, tailoring care to patients’ needs, understanding the patient as a unique human being, etc.) and lack of definition derived from patients During interviews, participants were asked what they valued in care, what they thought patient-centred care meant, and what constitutes patient-centred care; themes emerged inductively	• Relationship: Helpful, respectful, open communication • Decision-making: Being involved in decisions and to contribute in care consultations	–
Bann 2010 USA [35]	Survey of patients (*n* = 216; 43% aged 55 or greater; 184 women, 31 men) 85.2% women	To assess views about PCC among complementary and alternative medicine patients	patient-centered	Authors defined PCC as building an empathetic relationship that considers the patient as a partner with the health care provider in the priorities, problems, and goals of Treatment. The survey included 10 PCC attributes: I feel seen and heard as a unique individual by my therapist; My therapist has a full picture of me as a unique individual; My therapist is really interested in finding and addressing my health problems; The root causes of my problems are identified by my therapist; The root causes of my problems are being treated by my therapist; The treatment is individualized for me at each session; My therapist receives feedback from my body that guides treatment; My therapist asks me for feedback from my body that guides treatment; I know what to expect during treatment sessions; My therapist teaches me ways to relieve symptoms myself	–	• Humanization: Feeling seen and heard as a unique individual; receiving individualized treatment • Problems are solved: The therapist being interested in finding and addressing their health problems
Davis 2008 Australia [36]	Survey of patients (*n* = 78; mean age 82 years; 72 women, 6 men) 92.3% women	To assess views about PCC among older patients recently discharged from a sub-acute setting	person-centred	Authors defined PCC as valuing people as individuals and as the person being the focal point in a partnership that is both respectful and reciprocal The survey included five dimensions: personalisation, empowerment, information, approachability and availability, and respect	• Information: Many felt that they were not being told what was going on, lack of communication between staff • Relationship: Respect was typically demonstrated by staff.	• Humanization: Treated as a whole person • Ease of contact: Majority found they were unable to locate nurses for assistance, and would like to speak with nurses and doctors more often

### Additional PCC elements

Although studies did not consistently address PCC domains according to the McCormack et al. framework, other elements relevant to PCC were addressed in nine (81.8%) of 11 studies (Table 1). For example, timely responses to patient requests helped to gain trust and security and continuation and coordination of care following treatment helped patients to feel cared for. Flexibility of scheduling allowed patients to plan their life around care and inflexibility of scheduling and limited appointment times led to patients feeling devalued. Several studies identified the theme of humanization, meaning feeling seen and heard as a person and receiving individualized communication and treatment that fits their personal needs. There were no differences in additional PCC elements between studies comprised primarily of women [31, 34–36] and other studies.

## Discussion

PCCW may attenuate the widespread gendered disparities in health care delivery and outcomes. However, this review revealed that there are no established frameworks of PCCW. Studies varied in how they described PCC, with little direct consideration of women’s unique needs and preferences because none of the studies included solely women, or analyzed or reported findings by gender. Moreover, none of the studies addressed all 6 domains of an established PCC framework, identifying specific gaps in how PCCW has been conceptualized or operationalized [25]. However, additional PCC elements emerged in 9 of 11 studies including timely responses, flexible scheduling, and humanized or individualized management.

These findings are novel because no prior work had conceptualized PCC specifically for women. While numerous frameworks for PCC are available, comparison of domains across those frameworks demonstrated variability [14, 15, 25]. Our study also showed that, while some PCC components in included studies matched those of the McCormack et al. framework [25], additional PCC elements emerged. Given that the McCormack et al. framework was developed for cancer patients [25], the variability in PCC domains across frameworks underscores that some PCC elements may be common to all patients, while others may be unique to specific conditions. This is consistent with the fact that PCC is meant to accommodate individual needs and preferences [10], which in part must be influenced by conditions or health care issues, and in part by individual characteristics. It stands to reason then that at least some aspects of PCC may be specific to women, although that was not evident in the included studies because there were no differences in PCC domains between studies comprised primarily of women [31, 34–36] and other studies. Experts agree that there may not be a global definition of PCC [37]. However, our findings underscore the paucity of research on PCCW.

Strengths of this study include the use of rigorous methods [20] including a comprehensive search of multiple databases, independent screening and data extraction, compliance with standards for the reporting of review [21] and for searching of electronic databases [22], and use of a PCC framework upon which to map PCC elements from included studies [25]. Several factors may limit the interpretation and application of the findings. Despite having conducted a comprehensive search of multiple databases we may not have identified all relevant studies, in part because our search was restricted to English-language studies and studies that used the label of patient- or person-centred (or centered) care. We did not search the grey literature given the methodological challenges that have been identified by others [23, 24]; as a result, important information may have been missed. Few studies were eligible and none included solely women, so the findings may not truly represent the views of women about PCC. Risk of bias of included studies was not assessed as this is not customary for a scoping review [20]. Although scoping reviews often include consultation with stakeholders to interpret the findings, this step was not done because studies were few and provided sparse details [20]. Our analysis relied on the McCormack et al. PCC framework [25], which may not be a universally recognized or accepted framework. Still, as a comprehensive PCC framework, it served as a basis from which to assess whether and how PCCW has been studied, evaluated or improved.

The purpose of this study was to identify and compare existing PCCW frameworks. Strikingly, despite research demonstrating disparities in care among women [1–6], and global advocacy for research to improve quality of care for women [7–9], no research has conceptualized PCCW. Hence, there is little guidance on what constitutes PCCW or how to evaluate and improve PCCW. Given the aging and ethnic diversity of the female population, rise in labour force participation, shift to marriage and childbearing later in life, increase in single parent households largely headed by women, women’s simultaneous roles as paid workers and caregivers, and gaps in earnings for women compared with men, the socio-economic implications of poor health care for women are profound [38]. Given these socio-economic implications, consideration of PCCW is urgently needed. To better address PCCW, and in so doing alleviate or mitigate the socio-economic factors that contribute to gendered inequities in health care quality, advocates in Canada and the United States have issued recommendations for health care reform that include a focus on women’s health; comprehensive, integrated programs and services meeting women’s unique needs across the lifespan; better provider training about women’s unique health needs and the differential effects of particular problems; vigorous public health leadership to shape the women’s health agenda, recognizing the social and economic context of their lives; consideration of gender and health in all government policies; developing and implementing guidelines that include specific evidence-based gender elements; and sharing information with women directly [38, 39].

This review revealed several pressing areas where future research is needed. Primary research is needed to explore the elements of PCC valued by women including across different conditions. More needs to be understood about the challenges faced by women and health care professionals in achieving PCC. Finally, research is needed to examine whether and how policies, guidelines and interventions could better promote and support PCCW so that more women receive it. In addition to primary research, much could be learned from published investigations of women’s health care preferences and experiences in various contexts. For example, research has explored patient-centred outcomes desired by women exposed to sexual trauma or intimate partner violence [40], and what is considered by women to be patient-centred maternity care [41].

## Conclusions

Despite international calls for strategies to reduce gendered disparities in health care delivery and outcomes, this scoping review identified no studies that employed or developed a PCCW framework, and no studies that involved solely women in exploring how to achieve PCCW. Thus primary research is needed to generate knowledge about PCCW models, processes, facilitators, challenges, interventions and impacts.

## Supplementary information

**Additional file 1.** MEDLINE search strategy.

## Data Availability

The dataset(s) supporting the conclusions of this article is (are) included within the article (and its additional file(s).

## Abbreviations

PCC: Patient-centered care
PCCW: Patient-centered care for women
PICO: Population, Intervention, Comparisons, Outcomes
PRISMA: Preferred Reporting Items for Systematic Reviews and Meta-Analysis
