# Supplementary material for: How is patient-centred care conceptualized in women’s health: a scoping review
Source: BMC Womens Health. 2019 Dec 10;19:156. doi: 10.1186/s12905-019-0852-9 (PMC6902460; doi:10.1186/s12905-019-0852-9)
Supplement: Supplementary file 1 — Additional file 1. MEDLINE search strategy. [file 12905_2019_852_MOESM1_ESM.docx]

Additional File 1. MEDLINE search strategy

Database: Ovid MEDLINE: Epub Ahead of Print, In-Process & Other Non-Indexed Citations, Ovid MEDLINE® Daily and Ovid MEDLINE® <1946-Present> Search Strategy:
--------------------------------------------------------------------------------
1     women's health/ (25422)
2     women/ (14247)
3     female/ (7835541)
4     1 or 2 or 3 (7839777)
5     patient satisfaction/ (71947)
6     personal satisfaction/ (15404)
7     Patient Preference/ (5969)
8     Patient-Centered Care/ (15651)
9     (patient centered or patient-centered or patient centred or patient-centred).mp. (27001)
10     (person centered or person-centered or person centred or person-centred).mp. (3883)
11     (wom#n centered or wom#n-centered or wom#n centred or wom#n-centred).mp. (450)
12     professional-patient relations/ (24731)
13     Health Communication/ (1437)
14     Health Equity/ (367)
15     Health Services Accessibility/ (63814)
16     Patient Participation/ (22042)
17     5 or 6 or 7 or 8 or 9 or 10 or 11 or 12 or 13 or 14 or 15 or 16 (220827)
18     4 and 17 (110430)
19     limit 18 to (english language and yr="2008 -Current" and "all adult (19 plus years)") (50343)
20     limit 19 to (comment or editorial or interview or lectures or letter or news) (493)
21     19 not 20 (49850)
22     8 or 9 or 10 or 11 (30272)
23     4 and 22 (8723)
24     limit 23 to (english language and yr="2008 -Current" and "all adult (19 plus years)") (5055)
25     limit 24 to (comment or editorial or interview or lectures or letter or news) (26)
26     24 not 25 (5029)
27     depression/ (99502)
28     26 and 27 (161)
29     cardiac rehabilitation/ (1535)
30     Cardiovascular Diseases/ (128523)
31     26 and 29 (4)
32     26 and 30 (60)
33     family planning services/ or reproductive health services/ (25063)
34     26 and 33 (28)
35     Preventive Health Services/ (12323)
36     Health Promotion/ (65178)
37     Healthy Lifestyle/ (499)
38     35 or 36 or 37 (76434)
39     26 and 38 (116)
40     27 or 28 or 29 or 30 or 31 or 32 or 33 or 34 or 35 or 36 or 37 or 38 or 39 (325594)
41     26 not 40 (4669)
